# A novel pigeon paramyxovirus type 1 isolated from a sick racing pigeon in the Qinghai–Tibet Plateau of China shows high virulence in chickens

**DOI:** 10.17221/15/2024-VETMED

**Published:** 2024-11-28

**Authors:** Lina Tong, Xiaolong Gao, Ling Feng, Dongliang Yao, Xueqi Zhang, Yinggui Du, Yiyi Zhou, Fuhui Chen

**Affiliations:** College of Agriculture and Animal Husbandry, Qinghai University, Xining, P.R. China

**Keywords:** genotype XX, PPMV-1, velogenic strain

## Abstract

Pigeon paramyxovirus type-1 (PPMV-1) is the causative agent of pigeon Newcastle disease (ND), which has caused huge losses to the pigeon industry. In this study, a PPMV-1 strain, PPMV-1/QH-01/CH/23, was isolated from a sick racing pigeon in the Qinghai–Tibet Plateau, China in 2023. The mean death time of chicken embryos and the intracerebral pathogenicity index (ICPI) were 76.8 h and 1.25, indicating a mesogenic strain. Pigeon morbidity and mortality were 100% and 80%, respectively, and both were 80% in chickens; therefore, this isolate was velogenic for both pigeons and chickens. The fusion gene was amplified and sequenced for phylogenetic analysis, and the results indicated that the isolated strain possessed a virulent fusion protein cleavage site motif, ^112^R-R-Q-K-R-F^117^, and belonged to genotype XX (former sub-genotype VIc) of class II; this was different from the predominant sub-genotype, VI.2.1.1.2.2, which is prevalent in pigeons. To the best of our knowledge, this is the first report of a novel genotype XX isolate possessing high virulence for both chickens and pigeons in the Qinghai–Tibet Plateau, China.

Pigeon Newcastle disease (ND) is a highly contagious avian disease that has caused huge economic losses to the pigeon breeding industry (Kim et al. 2008). ND is caused by the virulent Newcastle disease virus (NDV), which can infect over 200 avian species (Kuhn et al. 2019). According to an updated unified phylogenetic classification system, NDVs were divided into two classes, class I and class II (Czegledi et al. 2006; Dimitrov et al. 2019). Each class contained multiple genotypes (Miller et al. 2010). NDV isolates of class I were classified as a single genotype (genotype 1) containing three sub-genotypes, whereas class II contained 20 distinct genotypes and multiple sub-genotypes (Dimitrov et al. 2019). Pigeon paramyxovirus type 1 (PPMV-1), an antigenic variant of NDV, is the causative agent of pigeon ND (Chong et al. 2013). The earliest PPMV-1 strain was isolated in Iraq in 1978; since then, the disease has rapidly spread into many countries (Kaleta et al. 1985). PPMV-1 caused the third ND pandemic, which resulted in mass flock mortality (Alexander et al. 1985; Hicks et al. 2019). In China, PPMV-1 circulated in several provinces and has been regularly recorded since 1985. For example, 14 pigeon-origin NDV isolates were obtained from sick pigeons in China between 1996 and 2005, and phylogenetic analysis revealed that most of the isolates were clustered into genotype VIb (now sub-genotype VI.2.1.1.2.2) (Liu et al. 2006). Tian et al. (2020) characterised 10 PPMV-1 viruses isolated from pigeons in China during 1996–2019 and found that genotype VI, especially sub-genotype VI.2.1.1.2.2, was predominant in pigeons. Zhan et al. (2022) isolated 21 PPMV-1 strains from diseased pigeons in China during 2007–2019, and phylogenetic analysis revealed that all isolates belonged to genotype VI, with most isolates classified under sub-genotype VI.2.1.1.2.2, indicating that VI.2.1.1.2.2 has become a prevalent genotype in pigeons in China. Genotype XX viruses, which contain some of the oldest known NDV isolates, were previously assigned as sub-genotype Vic. To date, most reported genotype XX viruses were isolated from chickens (Dimitrov et al. 2019), and strains from pigeons and other wild birds were relatively rare.

The PPMV-1 genome length is 15 192 nt, and the motif at fusion (F) protein cleavage site (Fcs) is ^112^GRQKRF^117^, ^112^RRKKRF^117^, or ^112^RRQKRF^117^, which are typical characteristics of virulent strains. However, most strains were usually mesogenic or lentogenic in chickens (Collins et al. 1994; Dortmans et al. 2010). Through serial passage in chickens, PPMV-1 could enhance its pathogenicity by introducing mutations in the virus genome (Dortmans et al. 2011). However, naturally isolated PPMV-1 with high virulence in chickens is rare.

In this study, an NDV strain was isolated from a sick racing pigeon in the Qinghai–Tibet Plateau, China, in 2023. Pathogenicity results showed that this strain was velogenic in chickens and pigeons, and phylogenetic analysis revealed that it belonged to genotype XX. This study enhances our understanding of PPMV-1 epidemiology.

## MATERIAL AND METHODS

During an epidemiological investigation of ND in the Qinghai–Tibet Plateau, China, a sick racing pigeon at a pigeon farm exhibited neurological signs of paralysis and torticollis, similar to symptoms of ND. Pigeons in this farm were immunised with live NDV vaccine (strain La Sota; Wuhan Keqian Biology Co., Ltd., Wuhan, P.R. China) by drinking water at 7 days of age, but the vaccination effect was not evaluated and no booster vaccination was administered. To determine the cause of the disease, tracheal and cloacal swabs were collected for NDV detection by reverse transcription-polymerase chain reaction (RT-PCR) with specific primers that targeted the *F* gene of NDV, as previously described (Ke et al. 2010). The PCR program was as follows: 94 °C for 2 min; 33 cycles of denaturation at 94 °C for 30 s, annealing at 55 °C for 30 s, an extension at 72 °C for 15 s, and a final extension at 72 °C for 5 minutes. The PCR products were visualised by 1% agarose gel electrophoresis. The NDV-positive swabs were soaked in phosphate-buffered saline (PBS) and treated with 10 000 IU penicillin–streptomycin overnight at 4 °C. After centrifugation, the supernatant was inoculated into the allantoic cavity of 9- to 11-day-old specific-pathogen-free (SPF) chicken embryos (Jinan Sais Poultry Co., Ltd., Jinan, P.R. China) for virus isolation. The harvested allantoic fluid was identified by the hemagglutination assay (HA) and hemagglutination-inhibition (HI) tests with specific antisera of H5, H7 and H9 subtypes of avian influenza virus (AIV) and NDV (Lijian Bio-Tech Co., Ltd., Qingdao, P.R. China) (Alexander 2009). The isolated virus was further plaque-purified on DF-1 cells as described by Zhang et al. (2022).

The mean death time (MDT) in 10-day-old chicken embryos and the intracerebral pathogenicity index (ICPI) in 1-day-old SPF chickens were measured according to a standard procedure (WOAH 2008). The criteria for pathogenicity were as follows: MDT: velogenic (< 60 h), mesogenic (60–90 h), or lentogenic (> 90 h); and ICPI: velogenic (1.50–2.00), mesogenic (0.70–1.50), or lentogenic (< 0.70).

In addition, twenty 2-week-old SPF chickens (Jinan Sais Poultry Co., Ltd., Jinan, P.R. China) were arbitrarily divided into two groups (10 per group). The first group was inoculated with 10^6^ EID_50_/0.1 ml of the QH-01 strain via the oculonasal route; the second group was inoculated with 100 μl PBS via the same route as a negative control. Moreover, 20 NDV-negative (without HI antibodies against NDV) healthy 4-week-old pigeons were divided into two groups (10 per group) and received the same treatment as did the chickens. All chickens and pigeons were raised individually for 14 days, and clinical signs, morbidity and mortality were observed or recorded post-infection. Animal experiments were performed in strict accordance with Animal Ethics Procedures and Guidelines of the Ministry of Health in China and the ARRIVE guidelines. All experimental procedures were approved and supervised by the Ethics Committee for the Care and Use of Laboratory Animals in Qinghai University, China (SL-2023048).

To investigate the evolutionary relationship between the isolated strain and other NDVs, the complete *F* gene was amplified and sequenced. Briefly, total RNA was extracted with RNAiso Plus (TakaRa Biotechnology, Dalian, P.R. China) from 700 μl allantoic fluid, and cDNA was synthesised with random primers using the PrimeScript^TM^ 1^st^ Strand cDNA Synthesis Kit (TakaRa Biotechnology, Dalian, P.R. China) according to the manufacturer’s instructions. The *F* gene was amplified using a pair of specific primers: F-Forward: 5'-TCATCGCGACACTAGGCAAC-3'; F-Reverse: 5'-TCTCCAACCGTTCTACCCGT-3' (designed in the current study). The program was as follows: 94 °C for 2 min; 33 cycles of denaturation at 94 °C for 30 s, annealing at 55 °C for 30 s, an extension at 72 °C for 60 s, and a final extension at 72 °C for 5 minutes. The PCR product was sequenced by Sangon Biotech Co., Ltd. (Shanghai, P.R. China). The nucleotide sequence analysis was conducted with DNASTAR (v3.1; DNASTAR, Madison, WI, USA). Then, a dataset containing the complete *F* gene coding nucleotide sequences of NDVs was downloaded from the GitHub repository (github.com/NDVconsortium/NDV_Sequence_Datasets) to perform the phylogenetic analysis. A maximum likelihood tree of class II NDVs was contrasted using the Molecular Evolutionary Genetics Analysis v11 (MEGA 11) based on the General Time Reversible model with 1 000 bootstrap replicates.

## RESULTS

The swabs collected from a sick pigeon with paralysis and torticollis were confirmed to be NDV-positive by RT-PCR (data not shown). The virus tested positive for HA, and hemagglutination was specifically inhibited by the NDV-specific antiserum but not by AIV-specific antisera (H5, H7, and H9). This isolate was further plaque-purified on DF-1 cells and named PPMV-1/QH-01/CH/23 (abbreviated as QH-01).

The MDT in 10-day-old chicken embryos exposed to the QH-01 strain was 76.8 h (60–90 h) and the ICPI in 1-day-old SPF chickens was 1.25 (0.70–1.50). According to the pathogenicity criteria, it was a mesogenic strain.

**Figure 1:**
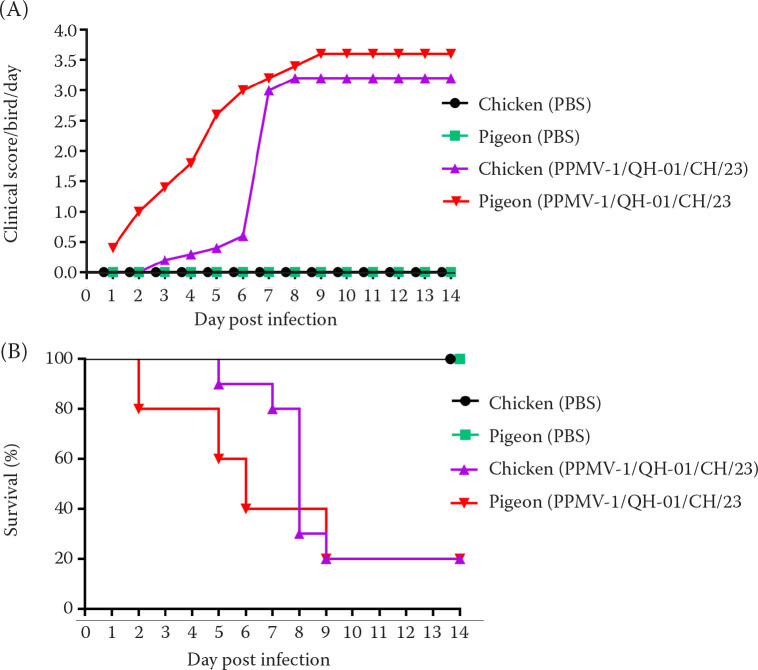
Morbidity and mortality of 2-week-old SPF chickens and 4-week-old NDV negative healthy pigeons infected with PPMV-1/QH-01/CH/23 (A) Clinical scores: 0 – normal; 1 – sick; 2 – paralysis/torticollis/wing drop/incoordination; 3 – prostration; 4 – death. (B) Survival curve

Figure 1 shows that 8/10 infected chickens developed severe clinical symptoms, such as diarrhoea, breathing difficulties, and wing drop, and eventually died, but the other two infected chickens showed no clinical symptoms and lived, similar to the negative groups (chickens and pigeons inoculated with PBS). All infected pigeons (10/10) developed obvious clinical symptoms, especially nervous signs such as paralysis, wing drop, incoordination and prostration, and eight pigeons eventually died, whereas the other two pigeons exhibited torticollis until the end of the experiment. The morbidity and mortality in 2-week-old chickens were both 80%, but were 100% and 80% in 4-week-old pigeons, respectively. These results indicated that the QH-01 strain was virulent for both chickens and pigeons.

**Figure 2:**
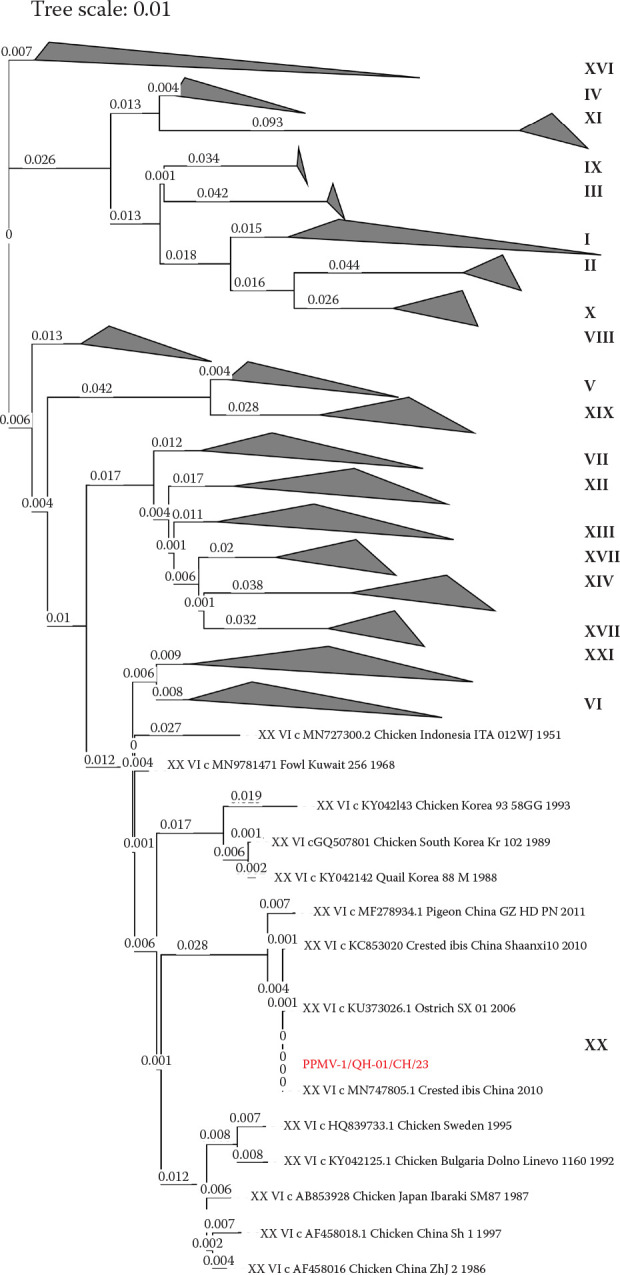
Phylogenetic analysis based on the complete *F* gene coding nucleotide sequences A maximum-likelihood tree of class II was constructed using the MEGA 11. For simplicity, except for genotype XX viruses (*n* = 15), all other genotypes are collapsed. The isolate PPMV-1/QH-01/CH/23 is highlighted in red colour

The complete *F*-gene coding sequence of the QH-01 isolate was obtained and analysed (submitted to GenBank and available under accession number PQ043262). The F protein contained 553 amino acids and the amino acids at Fcs were ^112^RRQKRF^117^, which is a typical characteristic of virulent strains; this was consistent with the above pathogenicity index and animal experiment results. Figure 2 shows that the QH-01 strain belonged to genotype XX (former sub-genotype VIc) of class II and was genetically close to the lentogenic NDV strain Ostrich/SX-01/06 and the mesogenic strain Crested ibis/China/10, but distant from the prevalent genotype VI strains in pigeons and the traditional vaccine strains.

## DISCUSSION

Pigeon ND has caused substantial losses to the pigeon breeding industry. It is caused by PPMV-1, a host variant of classical genotype VI chicken NDV that adapted in pigeons. In this study, a racing pigeon at a pigeon farm in the Qinghai–Tibet Plateau, China exhibited paralysis and torticollis, which are nervous signs often seen in neurotropic velogenic NDV-infected chickens. To identify the cause of the disease, tracheal and cloacal swabs were collected from this pigeon for RT-PCR identification and virus isolation. Finally, the samples were confirmed to be NDV-positive, and a PPMV-1 strain, QH-01, was isolated and characterised. Based on the Fcs motif, all PPMV-1 strains were considered virulent in poultry; however, these isolates had moderate or low pathogenicity for chickens, and some of them even had no virulence, as assessed through ICPI, in chickens. However, the present isolated QH-01 strain was highly virulent for both chickens and pigeons. Similarly, Nooruzzaman et al. (2021) reported that a PPMV-1 strain from Bangladesh induced high mortality in chickens and speculated that the strain might have originated in chickens. In our animal infection experiments, chickens had a slower disease onset compared with pigeons (Figure 1), probably due to differences in adaptation to the host immune system leading to slower viral entry and replication in chickens. Generally, the virulence of PPMV-1 viruses increases after serial passage in chickens (Dortmans et al. 2011). Therefore, whether the virulence of the QH-01 strain increases in chickens after passage needs further investigation.

Most of the PPMV-1 isolates belonged to genotype VI or genotype XXI (former sub-genotypes VIi, VIg, and VIm), and the predominant viruses circulating in pigeons was mainly sub-genotype VI.2.1.1.2.2 (former sub-genotype VIb) in China. We isolated and characterised strain PPMV-1/QH-01/CH/23, which belonged to genotype XX (former sub-genotype VIc) of class II. In this genotype, the oldest strain, chicken/Indonesia/ITA/012WJ, was collected from chickens in West Java, Indonesia in 1951 (Pandarangga et al. 2020). Therefore, the isolate in our study probably descended from chicken/Indonesia/ITA/012WJ, which is consistent with the previous speculation that PPMV-1 viruses originated from ancient chicken ancestors of genotype XX (Afonso 2021). Figure 2 shows that the two strains closest to QH-01 were Ostrich/SX-01/06 and Crested ibis/China/10, which indicated that these three viruses may have originated from the same host. However, Ostrich/SX-01/06 is lentogenic and Crested ibis/China/10 is mesogenic, which indicated that the amino acid at Fcs is not the only determinant of virulence and other factors, such as the HN protein or viral replication complex, could also influence PPMV-1 virulence (Dortmans et al. 2011; Chen et al. 2021). Consequently, the virulence factors need to be confirmed in future studies.

In this study, a genotype XX strain, PPMV-1/QH-01/CH/23, was isolated from a sick racing pigeon in the Qinghai–Tibet Plateau, China in 2023. The virus had multiple basic amino acids at Fcs and was highly virulent for both chickens and pigeons. To our knowledge, this is the first identification of a novel genotype XX isolate possessing high virulence for both chickens and pigeons in the Qinghai–Tibet Plateau, China, and these results emphasise the importance of sustained ND surveillance in pigeons.
